# COVID-19 pandemic: filling the gaps in psychiatry residency programs

**DOI:** 10.1590/2237-6089-2020-0051

**Published:** 2020-08-14

**Authors:** Carolina Barros Ferreira da Costa, Orli Carvalho da Silva

**Affiliations:** 1 Serviço de Psiquiatria e Psicologia Médica Hospital Clementino Fraga Filho UFRJ Rio de JaneiroRJ Brazil Serviço de Psiquiatria e Psicologia Médica , Hospital Clementino Fraga Filho (HUCFF/ UFRJ ), Rio de Janeiro , RJ , Brazil .; 2 Programa de Estudos e Assistência ao Uso Indevido de Drogas Instituto de Psiquiatria Universidade Federal do Rio de Janeiro Rio de JaneiroRJ Brazil Programa de Estudos e Assistência ao Uso Indevido de Drogas (PROJAD), Instituto de Psiquiatria (IPUB), Universidade Federal do Rio de Janeiro (UFRJ), Rio de Janeiro , RJ , Brazil .; 3 Instituto Nacional da Saúde da Mulher, da Criança e do Adolescente Fernandes Figueira Fundação Oswaldo Cruz Rio de JaneiroRJ Brazil Instituto Nacional da Saúde da Mulher, da Criança e do Adolescente Fernandes Figueira - Fundação Oswaldo Cruz (IFF/Fiocruz), Rio de Janeiro , RJ , Brazil .

Learning and practice are the best words to define a medical residency program. ^1^ During the COVID-19 pandemic, most residents had their programs reorganized in order to account for the great demand at the frontlines, as demonstrated by the preliminary report from the Brazilian Association for Medical Education (Associação Brasileira de Educação Médica [ABEM]). ^2^ Despite this reorganization, learning and practice in psychiatry have never been so intense and challenging, since mental health and the traditional ways of addressing it have been widely affected. ^3 - 5^

Throughout history, few events have shaped our society as have infectious disease outbreaks. The last catastrophic pandemic occurred a century ago, right before the recognition of psychiatry as a clinical specialty. ^6^ Consequently, only recent outbreaks such as Zika, Middle East respiratory syndrome (MERS) and severe acute respiratory syndrome (SARS) have driven global attention towards the prevention and management of the psychological consequences of these diseases. ^3 , 5 , 6^ Mental health and HIV studies have taught us the importance of harm reduction practices as well as how depression, stigma, guilt and shame can influence adherence to life-saving treatments. ^6^ As of now, the magnitude and the acuteness of COVID-19 have demonstrated that psychological features are not only consequences but also determinants of adherence to hygiene practices and social distancing. ^7^

Recent literature suggests that appointments, clinical supervisions and group discussions can occur via videoconference, ^3 , 4^ allowing for continuing practice and learning and also caring for our residents’ mental health. ^1^ Essential topics to be emphasized are: best practices in telemental health, the impact of excessive use of technology on mental health, promotion of mental health, occupational mental health, domestic violence, suicide and self-harm risk assessment, crisis intervention, communication skills for delivering bad news, and motivational interviewing to manage substance use problems and to improve adherence to hygiene habits and psychotropic therapy as well. ^5 , 8^ This reframing unveils our need to include humanitarian emergencies in the medical residency programs. ^6^

Regarding inpatient care, it is well known that severe mental illness can hinder the maintenance of adequate hygiene practices. ^9^ This pandemic reasserts the urgent need to address body and soul as one during learning and practice. There is no physical health without mental health. ^10^ Therefore, providing at least one isolation room is mandatory even for psychiatry wards in general hospitals. The asylum structure should be reorganized to avoid over-crowding, so that both patients and health professionals are safeguarded.

In the outpatient setting, easy access to patient data with updated phone numbers has been crucial to reach out and advise patients remotely. The goals are to reduce the risk of treatment abandonment, guarantee medication supplies and manage mental health crises. ^3 - 5^ Furthermore, telephone outreach has also been an important way to educate patients about COVID-19 preventive measures.

Training in telemental health may be here to stay and it is an opportunity to improve access for underserved communities. However, a sensitive issue is the excessive use of technology during and after the pandemic, especially among children and adolescents, since social skills and hands-on exploration are important for their development and well-being. ^11^ Medical residency program supervisors should expect an increasing demand for all ages over the next months, not only because of the pandemic but also due to activities overly dependent on technology. ^6 , 12^

Regarding the support provided to health professionals, this pandemic is a great opportunity for psychiatrists to promote mental health, prevent psychiatric disorders and, therefore, improve their sense of self-efficacy at the frontline. ^5^ Psychiatric disorders are known to increase the chances of error at work, which now represents a risk not only for patients but also for the professionals themselves. ^8^ This is a challenge because promotion of mental health and occupational mental health are frequently left aside in psychiatry residency programs.

Considering the urgency to adapt the routines of medical residency programs to the current context, the COVID-19 pandemic has reaffirmed the role of psychiatry as a medical specialty and seals the link between infectious disease and mental health in both clinical practice and research. These unexpected gains fill many gaps in learning and practice for future psychiatrists.
